# Availability of resources for paediatric hearing care in a South African province

**DOI:** 10.4102/phcfm.v16i1.3952

**Published:** 2024-03-22

**Authors:** Mukovhe Phanguphangu, Khomotjo Kgare, Ashley Flynn, Sinelihle Kotelana, Siphesihle Mfeketo, Sinovuyo Njiva

**Affiliations:** 1 Department of Rehabilitative Science, Faculty of Health Sciences, University of Fort Hare, East London, South Africa

**Keywords:** early hearing detection and intervention, paediatric and/or infant, state hospitals, resources, availability

## Abstract

**Background:**

Unavailability of healthcare resources can lead to poor patient outcomes. The latter is true for infants with hearing loss and require early hearing detection and intervention (EHDI).

**Aim:**

To determine the availability and distribution of resources for EHDI in state hospitals in the Eastern Cape (EC) province, South Africa.

**Setting:**

Sixteen state hospitals (nine district, four regional and three tertiary hospitals).

**Methods:**

Descriptive cross-sectional survey completed between July 2022 and October 2022.

**Results:**

Thirteen hospitals had audiologists (*n* = 4) or speech therapists and audiologists (*n* = 9). Specific to equipment, 10 hospitals had a screening otoacoustic emissions or automated auditory brainstem response, 8 hospitals had diagnostic middle ear analysers and only 3 hospitals had diagnostic auditory brainstem response and/or auditory steady state response. Twelve hospitals did not have visual response audiometry (VRA) and 94% had no hearing aid verification systems. Budget allocations were uneven, with only 10 hospitals, i.e., 4 districts, all regional and 2 tertiary hospitals being allocated varying amounts. Subsequently, only 50% provided newborn hearing screening, 56% provided diagnostic evaluations and 14 hospitals fitted hearing aids.

**Conclusion:**

Results revealed a limited and uneven distribution of resources, which negatively impacted the provision of EHDI. Even distribution of healthcare resources and further research aimed at strengthening hearing health services is recommended as these could potentially improve equitable access to EHDI and the overall quality of healthcare provided.

**Contribution:**

This study highlights the need for even distribution of resources and strengthening of health systems, especially in the dawn of the National Health Insurance.

## Introduction

An estimated 6:1000 babies are born with permanent disabling hearing loss (HL) in South Africa.^1^ For children with undetected HL, critical developmental milestones for optimal language acquisition are forfeited.^2^ Persistent delays in speech and language acquisition in turn lead to poor scholastic performance and academic achievement,^3^ which often results in diminished vocational prospects,^4^ social isolation^5^ and an overall poor quality of life.^6^ In adults, HL is linked to poor vocational outcomes, economic hardship and unemployment.^4^ Early hearing detection and intervention (EHDI) is thus important to prevent the development of these negative impacts.^6^

Early hearing detection and intervention is a four-component package that includes: (1) early detection through screening, (2) diagnostic assessment to diagnose the HL, (3) amplification through hearing aid fitting and (4) (re)habilitation therapy together with speech therapists (ST). Previous research has shown that EHDI helps maximise children with HL’s access to sound and the hearing world,^7^ which is important for the development of communication abilities. Additionally, when intervention is provided in the first 6 months of life, children with HL can develop language in line with their normal-hearing peers.^8^ The prerequisite resources for the provision of EHDI per level of care, according to the Health Professions Council of South Africa (HPCSA) guidelines for planning speech therapy and audiology services,^9^ are outlined in Table 1.^9^ According to these HPCSA guidelines,^9^ as depicted in Table 1, all hospitals should have an audiology technician (AUT) to conduct the screening (EHDI) and implement intervention plans as planned by the audiologist. In addition to an AUT, an audiologist (AU) is also required to conduct diagnostic assessments, diagnose the HL and plan the treatment or management of HL, i.e. the selection and fitting of hearing aids and planning the aural rehabilitation. Additionally, ST are also required to provide aural rehabilitation with paediatrics (except hearing aid fitting and verification) and early communication intervention. Specific to equipment, the HPCSA guideline stipulates that hospitals and clinics should have an otoscope, screening otoacoustic emissions (OAE) and/or automated auditory brainstem response (AABR), diagnostic middle ear analyser with 1000 Hz probe tone, a diagnostic audiometric booth (at least 2 m × 2 m) and a diagnostic audiometer capable of performing visual response audiometry. In addition, hearing aids, fitting software and verification systems are also required to objectively verify hearing aid fittings.

**TABLE 1:** Audiology early hearing detection and intervention services package and resource requirement per level of care.

Level	Services	HCPs	Equipment
District hospital	OAE and/or AABR screening Otoscopic examination Immittance measures Pure tone audiometry Visual response audiometry Hearing aid fitting Aural (re)habilitation Hearing aid verification Referrals for advanced testing (ABR/ASSR) and cochlear implants and bone-anchored hearing aids	1 AU: 300 PDE or 1.5 AU: 300 PDE (rural contexts), 1 AUT, 1 ST: 300 PDE or 1.5 ST: 300 PDE (rural contexts) and 2 STT	Otoscope Diagnostic middle ear analyser Diagnostic booth (2 m × 2 m) Diagnostic audiometer with free field speakers Visual response audiometry system OAE and/or AABR screener HA verification system HA fitting system
Regional/general hospital	All district level services plus: Advanced testing (OAE, ABR and/or ASSR) CAPD Referrals for cochlear implants and bone-anchored hearing aids	-	All district level equipment plus: Diagnostic OAE Diagnostic ABR and/or ASSR
Tertiary/central hospital	All regional level services plus: Cochlear implantation, middle ear implants Bone-anchored hearing aids Specialist and complex disorder clinics	1 AU: 200 PDE or 1.5 AU: 300 PDE (rural contexts), 1 AUT, 1 ST: 200 PDE or 1.5 ST: 300 PDE (rural contexts) and 1 STT	All regional level equipment plus: Cochlear implant mapping equipment

*Source:* Balton S, Singh S, Van Wyk P, et al. Guideline for planning speech therapy and audiology services at all levels of health care. Health Professions Council of South Africa [homepage on the Internet]. 2024 [cited 2024 Jan 04]. Available from: https://www.hpcsa.co.za/Content/upload/professional_boards/slh/guidelines/guideline_planning_STA_services_at_all_levels_health_care.pdf

HCP, Healthcare professional; OAE, otoacoustic emissions; AABR, automated auditory brainstem response; ABR, auditory brainstem response; ASSR, auditory steady state response; AU, audiologist; PDE, patient day equivalent; AUT, audiology technician; CAPD, central auditory processing disorders; HA, hearing aid; STT, speech therapy technician/assistant.

While EHDI has proven long-term savings globally, as the early rehabilitation of paediatric HL prevents the development of the negative impact of HL, previous research has reported the lack of resources to provide EHDI in various provinces in South Africa.^10,11,12,13,14^ However, there is a paucity of the literature on the availability of resources to provide EHDI at state facilities in the Eastern Cape (EC) province. This lack of context-specific literature may negatively influence budget allocations for the provision of EHDI at state facilities, including employment of qualified clinical staff to provide quality healthcare, procurement of equipment for assessment and diagnosis of HL and procurement of assistive devices for HL rehabilitation.^6^

Currently, the EC province Department of Health has a dearth of audiology services, with only 20 hospitals providing these services from a total of 91 state hospitals.^15^ Against this backdrop, this study sought to assess the availability of resources to provide EHDI in the EC province, South Africa. Findings from this study will be useful to advocate for budget allocations for the provision of EHDI services within the EC public health system. In addition, these findings can be used to promote access and improve the quality of hearing healthcare provided within state hospitals in the Eastern Cape, South Africa. The study aimed to assess the availability of resources to provide EHDI services in state hospitals in the EC province. The primary endpoints of this study were to determine: (1) whether the hospitals provided EHDI services or not, (2) budget allocations and (3) the availability of qualified healthcare professionals, screening and diagnostic equipment and clinical protocols for providing EHDI in state hospitals in the EC province.

## Research methods and design

### Study design

This was a descriptive quantitative cross-sectional survey.^16,17^

### Setting

This study was conducted in the EC province, and state hospitals under the Department of Health were chosen to participate in this study. These hospitals were chosen as an estimated 88% of the Eastern Cape Provincial population access their healthcare at public hospitals.^6^ During the study period, a total of 29 state hospitals and 1 primary health care clinic in Nelson Mandela Bay provided audiology and speech therapy services.

### Study population and sampling strategy

The population for this study included AU and speech therapists and audiologists (STA) working in state hospitals in the EC province. Non-probability convenience sampling^17^ was used to select and recruit the most senior AU or STA to participate in the study. In addition, community service officers in audiology or speech therapy and audiology were also included in cases where these community service officers were the only audiologist or speech therapist and audiologist employed in the hospital. To be included in the study, the prospective participants had to be working in state hospitals in the EC and currently registered with the HPCSA as either an AU or an STA. As there were only 20 hospitals with audiology services during the study period, sample size calculation was not done, but all hospitals were recruited to participate in the study.

### Data collection

A previously developed questionnaire^10^ was adapted to include information regarding the budget allocations and demographic characteristics of each participant in this study and included sections on the demographic characteristics of the hospitals, whether the hospitals provided EHDI services, the type of EHDI services provided and the availability of resources to provide these services. The questionnaire mainly used objective close-ended questions with yes or no, to tick all that apply and single text box style questions. To ensure completion rate, the questionnaire was designed to take between 5 min and 10 min to complete.^17^ The questionnaire was uploaded on www.surveymonkey.com for participants to complete online, and an email invitation was sent to all prospective participants with a link to the survey.

#### Data analysis

Descriptive statistics in the form of frequency tables and percentages was used to analyse the data for this study. Frequency tables were used as they are one of the most fundamental methods for presenting descriptive statistics and allow the researcher to display enormous amounts of data in a clear and straightforward way.

### Ethical considerations

This study adhered to the World Medical Association’s Declaration of Helsinki ethical principles^18^ of conducting research with human participants. Ethical clearance was obtained from the University of Fort Hare’s Faculty of Health Sciences Human Research Ethics Committee (Ref: 100118-054), and permission to conduct the study was acquired from clinical managers/CEOs of hospitals prior to the data collection.

To ensure and maintain privacy of participants, respondents were asked not to write their names on any of the data collection form. Information was provided to all prospective participants in an email invitation with a link to the survey and a page that allowed them to give consent. To give consent, participants had to click on ‘Consent to Participate’ on the first page of the online survey, and if they chose not to give consent, they were asked to exit the survey.

## Results

Sixteen hospitals completed the survey, yielding a 73% response rate. Over half (56%) of the hospitals (*n* = 9/16) were classified as district hospitals, and overall, there were 53 speech therapists and audiologists employed in the state hospitals that took part in this study during the study period. Four hospitals, which included three district hospitals and one regional hospital, had audiologists only, while three (19%) district hospitals did not have audiologists. Table 2 summarises the demographic characteristics of the participating hospitals stratified by level of care.

**TABLE 2:** Participant demographic characteristics.

Demographic characteristics	DH (*N* = 9)	RH (*N* = 4)	TH (*N* = 3)	Total (*N* = 16)
**Region of the Eastern Cape**				
Eastern	5	2	1	8
Central	1	2	1	4
Western	3	0	1	4
**Setting**				
Rural	6	3	1	10
Urban	3	1	2	6
**Availability of human resources**				
Hospitals with AUs only	3	1	0	4
Hospitals with both STAs	3	3	3	9
Hospitals with STs only	3	0	0	3
**Total number of HCPs per level of care**				
Audiologist	6	8	11	25
Speech therapists and audiologist	0	2	8	10
Speech therapist	7	5	6	18
AUT	0	0	0	0
STT	0	0	0	0

DH, district hospital; RH, regional hospital; TH, tertiary hospital; AU, audiologist; STA, speech therapist and audiologist; ST, speech therapist; HCP, healthcare professional; AUT, audiology technician; STT, speech therapy technician/assistant.

With regard to the availability of audiological equipment to provide EHDI, only 10 (62%) hospitals had access to an OAE, AABR or combined OAE-AABR screener. For diagnostic purposes, all hospitals had an otoscope, but only half of the hospitals (50%, *n* = 8) had a diagnostic middle ear analyser capable of performing a 1000 Hz probe tone, whereas only a quarter (25%) had a visual response audiometry. All of these are required for paediatric assessments. While all hospitals had access to a diagnostic audiometer, this is not a requirement for EHDI with babies younger than 6 months as these require objective measures such as auditory brainstem response (ABR) or auditory steady state response (ASSR) to assess their hearing. Specific to diagnostic OAE and ABR, which are required for advanced audiometry at regional and tertiary hospitals, none of the regional hospitals had diagnostic OAE or ABR equipment. However, all three tertiary hospitals had functional ABR and/or ASSR testing equipment. Furthermore, while all hospitals are supposed to have an objective hearing aid verification system, only one hospital had a hearing aid verification system. Table 3 summarises the availability of audiological equipment to provide EHDI stratified by level of care.

**TABLE 3:** Availability of equipment to provide early hearing detection and intervention in state hospitals in the Eastern Cape.

Audiological equipment	Availability							
	DH (*N* = 9)		RH (*N* = 4)		TH (*N* = 3)		Total (*N* = 16)	
	Yes	No	Yes	No	Yes	No	Yes	No
Screening OAE, AABR or combined OAE and/or AABR	4	5	4	0	3	0	10	6
**Diagnostic equipment**								
Otoscope	9	0	4	0	3	0	16	0
Diagnostic middle ear analyser with 1000 Hz probe tone	4	5	1	3	3	0	8	8
Diagnostic clinical audiometer	6	3	4	0	3	0	13	3
Diagnostic OAE	N/A	-	0	4	2	1	2	5
Diagnostic ABR and/or ASSR	N/A	-	0	4	3	0	3	4
2 m × 2 m audiometric booth	4	5	3	1	3	0	10	6
Visual response audiometry	1	8	2	2	1	2	4	12
**Other equipment**								
Hearing aid verification	0	0	0	0	1	2	1	15

DH, district hospital; RH, regional hospital; TH, tertiary hospital; ABR, auditory brainstem response; ASSR, auditory steady state response; OAE, otoacoustic emissions; AABR, automated auditory brainstem response N/A.

**FIGURE 1:**
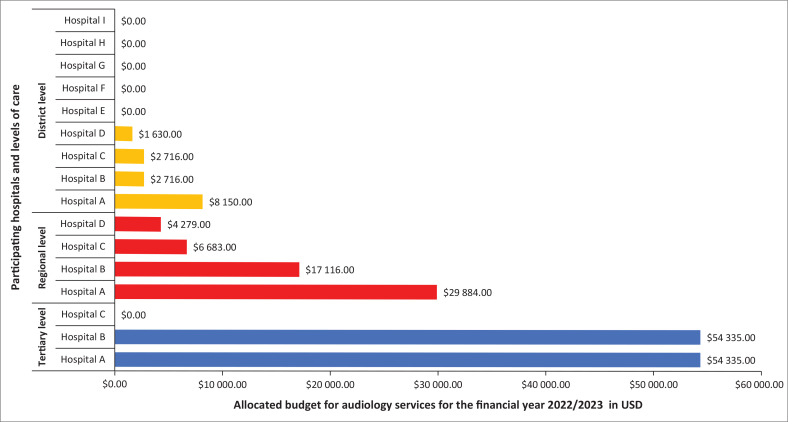
Budget allocations for audiology services in 2022–2023 in USD.

Budget allocations for the hospitals were provided locally by each hospitals’ finance department. Ten of the 16 hospitals were allocated budgets for the financial year 2022–23 to provide audiological services including EHDI. The budgets allocated were for the procurement of audiological equipment, amplification devices (hearing aids and cochlear implants) and consumables. Figure 1 shows the specific budgets allocated to each hospital in USD (as of 20 November 2023), stratified by hospital level of care. As can be noted in the figure, only four of the nine (44%) district hospitals were allocated a budget. The highest allocated budget for district hospitals was only $8150.00. While all regional hospitals were allocated budgets, these were very different and uneven, despite these hospitals being on the same level and expected to provide the same level of care. For instance, while one regional hospital was allocated a total of $4279.00, another was allocated $29 884.00. Furthermore, only two of the three tertiary hospitals were allocated budgets ($54 335.00 each), and yet, all the three hospitals were expected to provide advanced audiological and EHDI services, including expensive cochlear implantation services.

With regard to EHDI, only eight hospitals (50%) provided newborn hearing screening services, while 56% (*n* = 9) provided diagnostic hearing assessments. Only 13 hospitals with audiologists provided hearing aid fittings and only tertiary hospitals (*n* = 3) had cochlear implant services. Table 4 summarises the components of EHDI services provided by the hospitals stratified by level of care.

**TABLE 4:** Type of early hearing detection and intervention services provided in state hospital in the Eastern Cape province.

EHDI services provided	DH (*N* = 9)	RH (*N* = 4)	TH (*N* = 3)	Total (*N* = 16)	
				*n*	%
**Newborn hearing screening**					
Yes	2	3	3	8	50
No	7	1	0	8	50
**Type of screening**					
Universal	1	0	0	1	12
Risk-based screening	1	3	3	7	88
**Diagnostic hearing evaluation**					
Yes	3	3	3	9	56
No (refers to next level of care)	6	1	0	7	44
**Aural rehabilitation services**					
Hearing aid fittings	6	4	3	13	88
CI (tertiary service)	-	-	3	3	100
Aural rehabilitation including speech therapy	6	4	3	13	88

Note: One regional hospital did not perform hearing screening or hearing evaluation but provides hearing aid fittings and aural rehabilitation for patients down-referred from a higher level of care.

EHDI, early hearing detection and intervention; DH, district hospital; RH, regional hospital; TH, tertiary hospital; CI, cochlear implantation.

Respondents were also asked to list the clinical protocols they used to guide their clinical decision-making and implementation of services for newborn hearing screening, paediatric diagnostic evaluations and the provision of aural rehabilitation. Only 14 hospitals completed this section of the questionnaire, and the results are summarised in Table 5. Importantly, hospitals could list more than one protocol for each component of EHDI, e.g. one hospital could list the HPCSA guideline and internal standard operating procedures (SOP). As can be seen, over 50% of district hospitals had no protocols for hearing screening, diagnostic testing and aural rehabilitation.

**TABLE 5:** Protocols in the provision of early hearing detection and intervention services in state hospitals in the Eastern Cape.

Level of care	DH (*N* = 9)	RH (*N* = 4)	TH (*N* = 3)	Total (*N* = 16)
**Protocol/SOP/guideline**	-	-	-	-
**Hearing screening**				
No protocol	5	0	0	5
HPCSA guideline	2	3	2	7
JCIH guideline	1	1	0	2
Internal SOP	1	1	1	3
**Diagnostic testing**				
No protocol	6	1	0	7
Internal SOP	1	1	2	4
HPCSA guideline	2	2	1	5
**Aural rehabilitation**				
No protocol	7	1	1	9
Internal SOP	2	3	2	5
AAA guideline	0	1	0	1

DH, district hospital; RH, regional hospital; TH, tertiary hospital; AAA, American Academy of Audiology; JCIH, Joint Committee on Infant Hearing; HPCSA, Health Professions Council of South Africa; SOP, standard operating procedures.

Specific to challenges in providing EHDI, 10 participants reported that a shortage of staff limited their capacity to provide EHDI, as one of the participants stated, ‘it is often difficult to prioritise Newborn hearing screening when the OPD is fully booked, and you are the only Audiologist in the Hospital’. In addition, the management’s unwillingness to employ an additional audiologist further hindered service provision. A shortage of equipment was also identified as a challenge as five hospitals stated they were unable to perform screening because of lack of equipment.

## Discussion

As far as the researchers know, this was the first study to document the availability of resources to provide EHDI in state hospitals in the EC province, South Africa. Hospitals depend on the availability of resources such as screening and diagnostic audiological equipment, human resources in the form of audiologists, finances and clinical protocols to provide EHDI services.^14^ Thus, a lack of any of these resources can impede the provision of EHDI. Findings from this study showed an uneven distribution of the essential resources for providing EHDI within the state hospitals in the EC Province.

Findings from this study demonstrated a shortage and an uneven distribution of essential human resources for the provision of EHDI. As per the HPCSA, each hospital should have at least one audiologist, one AUT, one ST and one speech therapy technician or assistant (STT) per 300-patient day equivalence (PDE). Yet, none of the hospitals had an audiology technician or speech therapy assistant. Unfortunately, while the HPCSA guideline stipulates the employment of AUTs and STTs, none of these categories of professions exist on organograms in government hospitals in the EC, and there are no institutions in South Africa that provide training for these categories of professions, which may negatively impact service provision. Subsequently, the HPCSA is urged to reconsider the inclusion of AUT and STT professionals as required professionals in the provision of audiological services including EHDI, as there are no training institutions for these professional categories, and the positions for these professional categories do not exist in government hospitals. In addition to the lack of the AUT and STT professionals, 19% of the district level hospitals did not have audiologists while four (*n* = 4, 25%) hospitals did not have ST.

Unfortunately, patient day equivalent information per hospital was not available to estimate the PDE-HCP ratio and evaluate the equity of distribution of these resources. However, when considering the HPCSA guideline for planning speech therapy and audiology services per level of care, coupled with the fact that some hospitals had more than one HCP while others had none, it can be inferred that the distribution of these professionals was uneven. This shortage of human resources negatively impacted the provision of EHDI services in the EC as only eight hospitals (50%) in this study provided newborn hearing screening, which was mainly risk based (90%) instead of universal screening (10%). While this 50% coverage is higher than the previously reported 27% in a previous Gauteng study,^19^ the Gauteng study was conducted over a decade ago, and this coverage may have changed as the study was published. Furthermore, while 50% conducted the screening, only 10% of this screening was universal, which is against the World Health Organization recommendation that advocates for universal screening and thus for all newborns to be screened prior to hospital discharge.^20,21,22^ In addition, this poor coverage of universal newborn hearing screening means hospitals could be potentially missing a high number of babies born with congenital hearing loss but classified as low-risk as these would not be screened.

Although the main reason for a smaller coverage in the Gauteng study was the lack of appropriate equipment, the main reason reported in the present study was shortage or inadequate staff to provide screening. As one of the participants stated, ‘it is often difficult to prioritise Newborn hearing screening when the OPD is fully booked, and you are the only Audiologist in the Hospital’. These findings are similar to those reported in previous studies.^19,23,24,25^ A study in KwaZulu-Natal Province^25^ where participants argued that uneven distribution of audiologists and speech therapists remains a challenge in the provision of EHDI.^18,19,20,21,22^ Similarly, a study^25^ in the Gauteng province also reported that the insufficient number of audiologists available to provide screening affects the provision of early hearing detection and intervention services. Subsequently, we recommend equitable distribution of health professionals for the provision of early hearing detection and intervention in state hospitals in the EC, especially considering the dawn of the National Health Insurance, where babies should have equitable access to hearing health. We further recommend a horizontal equity approach to increase the capacity of state hospitals to provide early hearing detection and intervention services and reduce the burden of unidentified babies with hearing loss in the Eastern Cape province.

The recommendations for the equitable distribution of these professionals in state hospitals were also recommended by a previous study,^11^ which explored the national audiology practitioner-population in South Africa between 2002 and 2017 and found a 0.57:10 000 ratio of practitioner-population. In comparison to the national average, the Eastern Cape ratio is much lower at 0.19 per 10 000 population.^6^ As a result, most hospitals do not have the capacity to provide audiological services including EHDI, which may present challenges specifically considering the dawn of the National Health Insurance,^26^ which requires facilities to be fully equipped to provide a full package of audiological care. The creation of more job opportunities and an equitable distribution of audiologists within the state hospitals in the EC are recommended to increase the staff capacity to provide these services. In addition, contracted partnerships with private practitioners, as is the case in the United Kingdom, where private practices can form partnerships with the National Health Services to provide service, may offer a solution to increase access to EHDI, which is crucial for babies with HL. Furthermore, collaborative and transdisciplinary and task-shifting practices where nursing personnel can be trained to perform hearing screening within the labour and nursery wards and link these programmes to audiology departments are recommended as nurses spend more time with the patients and may thus be able to incorporate hearing screening into their nursing assessments within the wards. The latter has successfully reduced the workload on audiologists and increased access to EHDI for children with HL in the United States.^27,28^ An even distribution of resources is thus recommended to ensure more hospitals can provide EHDI.

To successfully implement EHDI, hospitals also depend on the availability of screening and diagnostic equipment. In the present study, only 10 hospitals (63%) had screening equipment, i.e. screening OAE or AABR to provide newborn hearing screening services and were providing these services. These findings are in contrast to those reported in a 2010 study^10^ in Gauteng, where all state hospitals (*n* = 11) had screening equipment, i.e. screening OAE or AABR. Specific to diagnostic assessments, a diagnostic middle ear analyser with 1000 Hz probe tone is recommended for tympanometry, while a diagnostic clinical audiometer coupled with VRA is required for subjective hearing testing. In the present study, almost two-thirds of the hospitals (*n* = 10, 63%) had diagnostic middle ear analyser with 1000 Hz probe tone, which is in contrast to the Gauteng study where only 37% of the hospitals had high-frequency tympanometry. However, while 81% (*n* = 13) had diagnostic clinical audiometers, only a quarter (*n* = 4, 25%) had visual response audiometry. This is in contrast to the findings of the Gauteng study, where 100% of the hospitals had diagnostic audiometers and 78% VRA. In addition to diagnostic audiometer and middle ear analyser, a 2 m × 2 m diagnostic audiometric booth is required for hearing evaluations, and in the present study, only 81% of the hospitals were equipped with a booth, in contrast with 100% in the Gauteng study. Per the HPCSA guideline, regional and tertiary hospitals should have diagnostic OAE and ABR or ASSR. In this study, 29% and 43% of the hospitals were equipped with diagnostic OAE and ABR/ASSR, respectively, which was higher than the 18% and 36% reported in the Gauteng study. However, the Gauteng study was conducted over a decade ago, and this the data may have changed.

Early hearing detection and intervention also requires financial resources for the procurement of equipment and amplification devices for the treatment of HL.^29^ In this study, 9 of the 16 hospitals were allocated varying amounts of money to render audiological services, including EHDI. However, these allocations were uneven, and as can be seen in Figure 1, only four (44%) of the nine district hospitals were allocated budgets varying from $1630.00 to $8150.00 (conversion from South African Rand on 20 November 2023). While all regional hospitals were allocated budgets, these were also uneven and varied from $4279.00 to $29 884.00 (conversation from South African Rand on 20 November 2023). Similarly, only two of the three tertiary hospitals were allocated budgets, totalling $54 335.00 each (conversion from South African Rand on 20 November 2023). Interestingly, while regional hospitals are supposed to provide testing such as ABR testing, two regional hospitals were allocated less budget than one district hospital. Likewise, while tertiary hospitals are expected to provide advanced and expensive services such as cochlear implantation and bone-anchored hearing aid fittings, one of the three hospitals was not allocated a budget.

Unfortunately, without adequate financial resources commensurate with the level of care and services expected, hospitals cannot procure the necessary equipment and assistive devices, which may reduce access and quality of healthcare provided.^6^ Therefore, we recommend a revision in the budget allocation process for audiology services in state hospitals in the EC, to ensure hospitals can provide high-quality audiological services, especially for services such as EHDI that aims to reduce the impact of unidentified HL.

The use of clinical protocols and SOPs is vital to ensure the provision of evidence-based care. In this study, participants were requested to list the clinical protocols they used to guide their decision-making process for screening, diagnostic testing and aural rehabilitation. The results showed that most district hospitals had no protocols for screening (*n* = 5), diagnostic testing (*n* = 6) and aural rehabilitation (*n* = 7). Regional and tertiary hospitals were equipped with clinical protocols or SOPs, even though their application was varied across institutions. For instance, while two regional hospitals used the HPCSA guidelines for performing diagnostic testing, one regional hospital used an internal SOP. Similarly, the protocols used for aural rehabilitation were varied, with seven of the nine district hospitals using no protocols, while two tertiary hospitals used the internal SOPs. These findings are similar to those reported in a study in Gauteng, where only 28% of departments reported having a protocol.^10^ This variation in the application of protocols utilised in clinical care has dire implications on the quality of care provided to patients. Clinical protocols and SOPS should be used in all areas of healthcare, including EHDI, as this will ensure that the healthcare provided is evidence based. In addition, the use of clinical protocols is important for reducing the risk of adverse events, ensuring the highest standards of clinical care provided to all patients and also assists in carryover of patient care in cases where staff retire or leave institutions.^29^ Subsequently, the development, distribution and implementation of SOPs in all hospitals in the EC is recommended to ensure that standardised quality of care is provided to all patients.

### Strengths and limitations

The study has strengths and limitations to consider. The high response rate and the use of an adapted version of a previously validated data collection tool minimise random error and selection bias. The comprehensive dataset without missing data ensures the accuracy of the data analysis and reporting. Furthermore, the inclusion of participants from all three levels of hospitals with good representation enhances the potential generalisability of the findings to other hospitals in similar levels of care within the EC province. In addition, the anonymity of the respondents may have also positively influenced the participants to respond truthfully in this study. The limitations of our study, however, include its cross-sectional and descriptive nature, having utilised an online platform to collect the data and relying on reports from one audiologist per hospital. This hinders the verification of answers from the respondents and thus affects the reliability of the data as some participants may have given false information for social desirability.

### Recommendations

The varied availability of resources for the provision of EHDI limits equitable access to early detection and intervention of HL for babies in the Eastern Cape, and this limited access to EHDI has negative implications on the prognosis of rehabilitation of children identified with HL. In addition, considering the dawn of the National Health Insurance in South Africa, this varied and unequitable distribution of resources has implications on the hospitals’ compliance with the Office of Health Standards and thus the accreditation for the provision of National Health Insurance services.

Based on these findings, the following recommendations were made for future practice and policy:

- A systematic and equitable distribution of HCPs for the provision of audiological services including EHDI per level of care.
- Centralised and equitable allocation of budgets to ensure equitable provision and access to EHDI for all babies in the EC Province.
- Development, distribution and implementation of protocols and SOPS for EHDI across state hospitals to ensure equitable and standardised level of care for all babies.
- The HPCSA should re-evaluate the recommendations for AUT and STT professionals in the provision of audiological services including EHDI, as there are no training institutions for these professional categories, and the positions for these professional categories do not exist in government hospitals.

Furthermore the following recommendations were made for future research:

- Health systems strengthening studies to ensure equitable access to early hearing detection and intervetnion services, especially given the dawn of the National Health Insurance in South Africa.
- Evaluation of the current status of EC state hospitals regarding the Joint Committee on Infant Hearing guidelines of the 1:2:3 EHDI Framework.
- Qualitative studies to understand the process for the distribution and allocation of resources for the provision of audiological care in the Eastern Cape province.

## Conclusion

Based on the findings of this study, it can be concluded that there was varied availability and an uneven distribution of healthcare resources for EHDI in state hospitals in the EC Province, South Africa. This variation in the distribution of resources negatively affected the provision of EHDI, and as a result, only 50% of the hospitals provided newborn screening which was mainly (88%) risk based. Additionally, only 56% of the hospitals provided diagnostic hearing evaluations, while 83% of the hospitals fitted hearing aids to patients who required hearing aid fittings. All three tertiary hospitals also provided cochlear implantation and mapping services in addition to hearing aid fittings.
